# Creating moral space: How military chaplains in the Netherlands support veterans with moral injury

**DOI:** 10.3389/fsoc.2025.1636853

**Published:** 2025-10-13

**Authors:** Laura Mudde, Carmen Schuhmann, Gaby Jacobs

**Affiliations:** University of Humanistic Studies, Research Group Humanist Chaplaincy Studies for a Plural Society, Utrecht, Netherlands

**Keywords:** moral injury, veterans, military chaplaincy, moral learning, longitudinal qualitative research

## Abstract

**Introduction:**

The concept of moral injury has drawn attention to the role of military chaplains (MCs) in supporting veterans. While international research has explored military chaplaincy interventions, collaboration with mental health professionals, and institutional influences, longitudinal, process-oriented insights into military chaplaincy practice remain scarce, particularly in the Dutch context, which combines secularization, pluralized worldviews, and a unique institutional position of MCs.

**Methods:**

This study employs a qualitative longitudinal design, analyzing the practice of 6 Dutch MCs in their counseling work with post-active veterans over a period of 3–12 months. Data consists of detailed conversation reports from each dyad and quarterly group reflections with participating MCs. Analysis included cross-sectional and longitudinal approaches, identifying both patterns in how MCs address moral injury in one-on-one meetings with veterans and how these meetings unfold over time.

**Results:**

Three interrelated dimensions characterize Dutch MCs' practice: presence – encompassing the physical setting and relational quality of encounters; their institutional position which we characterize as an embedded independent position within the military and healthcare systems; and moral reflection – referring to attuned pathways of how MCs address moral struggles. Longitudinal analysis revealed four typologies of contact development: (1) clear request and alignment, (2) emerging focus, (3) development through the relationship, and (4) minimal responsiveness with story-sharing focus. Breakthrough moments were observed primarily in types 1–3 and required sustained engagement.

**Discussion:**

The study emphasizes the moral significance of relational presence and the embedded independent position of MCs in addressing moral injury. Dutch MCs create a ‘moral space' allowing veterans to explore moral concerns, re-establish self-trust, and recognize broader sociopolitical dimensions of moral injury. These findings highlight the importance of attuned, politicized, and confidential care in addressing moral injury, with implications for professional development, interdisciplinary collaboration, and the quality of veteran care.

## Introduction

The long-term consequences of war in veterans' lives can include profound moral struggles, related to questions about whether they have done enough, or whether they did the right thing. While these moral struggles are as old as war itself, the concept of *moral injury* denoting these struggles was introduced in the 1990s, by psychiatrist Jonathan Shay. Moral injury refers to a specific type of struggles caused by severe violations of personal moral values, either by one's own actions or those of a legitimate authority (Shay, 1994). Since its further conceptual development by Litz et al. (2009), moral injury has attracted growing interdisciplinary interest, particularly in relation to veterans (Koenig and Al Zaben, 2021). The increased attention has also highlighted the role of military chaplains (MCs) in helping veterans address such moral struggles.

MCs have long provided spiritual and moral guidance within military organizations. While military chaplaincy exists in most Western armed forces, the roles and positions of military chaplaincy differ across countries. MCs may be embedded in military units or operate from a parish; they may serve a specific faith tradition or take a multi- or interfaith approach; also, the spectrum of worldviews represented varies (Bock, 1998; Liuski and Ubani, 2020; Michalowski, 2015). These variations are shaped not only by the religious landscape of the respective countries, but also by institutional arrangements making military chaplaincy a diverse and context-dependent field (Pleizier and Schuhmann, 2022). This article explores how MCs engage with moral injury in their one-on-one work with post-active veterans in the Dutch context in the Netherlands.

The Dutch context provides a distinctive perspective. Culturally, it represents a highly secularized society characterized by the pluralization of worldviews and the privatization of religion (Bernts and Berghuijs, 2016). These dynamics have shaped the Dutch chaplaincy landscape—for example, by the inclusion of humanist MCs in the Dutch Armed Forces since 1964^1^ and a broader move toward interreligious and worldview-inclusive approaches (Schuhmann et al., 2021). Institutionally, Dutch MCs are formally employed by the Ministry of Defence but maintain civilian status and are affiliated with religious or humanist sending institutions. This dual positioning – often described as having “*one foot inside and one foot outside the military*” (Schuhmann et al., 2023, p. 617) – enables MCs to be both embedded in military life and at the same time offer a sanctuary for reflection. Together, these cultural and organizational dynamics offer a unique context for studying how MCs address moral injury.

If we look at the existing empirical research on MCs and moral injury in the international context, three main lines of inquiry can be observed. First, a focus on evaluating specific chaplaincy interventions, such as adaptations of existing therapeutic models (e.g., *Cognitive Processing Therapy*: Koenig et al., 2017; Pearce et al., 2018) or chaplain-specific interventions like *Pastoral Narrative Disclosure* (Carey and Hodgson, 2018; Carey et al., 2024) and *Acceptance and Forgiveness Therapy* (Pernicano et al., 2022). These studies are mostly small-scale, quantitative, and situated in North American or Australian contexts (Jones et al., 2022).

Second, a related line of research examines collaborative programs between MCs and mental health professionals, such as *Building Spiritual Strength* (Usset et al., 2021) and the *Search for Meaning* program (Angel et al., 2023). These programs integrate psychoeducation and moral reflection to address PTSD and moral injury, again largely within the North American context.

Third, recent studies from European settings explore how institutional and cultural conditions shape military chaplaincy practices. These studies reveal how contextual factors shape military chaplaincy, underscore the significance of relational trust, and highlight how MCs offer veterans a place to explore and reconstruct veterans' moral worldviews (Grimell, 2022, 2024a; Liuski and Ubani, 2021; Pleizier and Schuhmann, 2022). A core feature of military chaplaincy practice that has been identified in European and NATO contexts, is the ministry of presence (Grimell et al., 2025). This term refers to a MCs' intentional availability and attentiveness to another person, without predefined goals or time constraints, and is also recognized as a hallmark of general chaplaincy practice (Adams, 2019; Lawton and Cadge, 2024). Notably, most of these studies are based on retrospective interviews in which chaplains reflect on their general practice.

### Aim

The aim of this study is to gain more sight into the Dutch military chaplaincy practice in supporting veterans with moral injury. These insights can support MCs in reflecting on and developing their own professional roles, lead to more effective interdisciplinary collaboration with MCs, and ultimately contribute to the quality of care for veterans. This is particularly urgent given the serious consequences of unresolved moral injury, including social withdrawal and increased risk of suicide (Jamieson et al., 2023; Smigelsky et al., 2024).

Whereas existing studies often evaluate predefined interventions or rely on retrospective data, this study takes a qualitative, longitudinal approach, based on detailed reports by MCs of their actual one-on-one encounters with veterans.^2^ The study is guided by the following question:

- How do Dutch MCs address the moral dimensions of moral injury in one-on-one meetings with post-active veterans over time?

We position this article within research into the professional chaplaincy practice aiming to describe it in terms of both ‘what' chaplains do and ‘to what end' they do this (Massey et al., 2015). This reflects an understanding of the chaplaincy practice as being both process- and outcome-oriented (Damen et al., 2020; Smit et al., 2024). On the one hand, the actions of MCs in one-on-one conversations unfold over time within a relational context (process-orientation). Simultaneously, these actions are guided by notions of what would be ‘change for the better' in the specific situation (outcome-orientation). This line of thinking moreover, provides space for a grounded analysis of the professional practice of MCs in which actions, value orientation *and* contextual positioning of chaplains are examined in relation to each other (see also Den Toom et al., 2023).

In what follows, we first elaborate on the specific character of Dutch military chaplaincy. We then outline the research methods, participants, and ethical considerations, followed by the presentation of our findings and a discussion of the findings.

### Military chaplaincy in the Dutch context

In the Netherlands, military chaplaincy is primarily available to veterans, military personnel, and civilian staff of the Dutch armed forces, and secondarily to their family members. In addition, contributing to the moral climate of the armed forces more broadly is also considered part of the MCs' professional mandate. Dutch MCs fulfill three core roles: they offer spiritual counseling during military training, on deployment, and to post-active veterans; they lead religious services, ceremonies, and rituals; and they contribute to moral education within military training programs.^3^ As a result, service members may encounter MCs throughout their entire military career. This article focuses specifically on the role of MCs in spiritual counseling in the context of moral injury among post-active veterans—typically offered through one-on-one meetings initiated by the veteran.

In line with the Netherlands' plural religious and spiritual landscape, Dutch military chaplaincy formally includes eight traditions: Protestant, Roman Catholic, Humanist, Muslim, Jewish, Hindu, Buddhist and, since 2024, Orthodox.^4^ While other sectors of Dutch chaplaincy (e.g., healthcare or ambulant care) include chaplains who are not affiliated with any religious or spiritual sending institution (Napel-Roos et al., 2021), this is not the case within the Ministry of Defence. Dutch MCs are always affiliated with a sending institution which informs their theological or philosophical education and continues to serve as a reference point throughout their careers. As a result, MCs operate within two sets of professional guidelines: those of the Ministry of Defence and those of their sending institution. However, despite their formal affiliations, many MCs adopt interreligious or worldview-inclusive approaches in practice (van Dijk, 2019; Schuhmann et al., 2023). This can also be attributed to the fact that the work of MCs who meet post-active veterans is organized regionally rather than denominationally.

Dutch MCs have a unique institutional position^5^ which significantly shapes how they perform their different tasks. As already mentioned, Dutch MCs often describe their role as having “*one foot inside and one foot outside the military”* (Schuhmann et al., 2023, p. 617). The “foot outside” refers to their civilian status: although employed by the Ministry of Defence, they do not hold military command responsibilities and are instead formally accountable to the worldview-based institutions they represent. Their military rank (typically equivalent to captain, major, or lieutenant-colonel) is symbolic, underscoring their embeddedness without implying command authority. This unique position allows MCs to provide sanctuary to service members and veterans, offering a trusted space for reflection on deeply personal, moral, or spiritual concerns—even those that may challenge prevailing military values. This structure reflects the broader Dutch principle of church–state separation, which ensures mutual autonomy: religious institutions do not interfere in state affairs, and vice versa (van Dijk, 2019).

At the same time, MCs also have a “foot inside the military”: they are closely integrated into daily military life. Dutch military chaplaincy is rooted in ‘presence': the MCs walk around at the base, they partake in sport activities, they travel with soldiers to participate in training camps and they join the unit for deployment abroad. “*Without being a soldier, they live a soldier's life*” (Pleizier and Schuhmann, 2022, p. 5). This embeddedness allows them to understand military life from the inside out.

To be eligible to work as a MC for the Dutch Ministry of Defence, candidates must hold a master's degree from a religious or humanist university. In addition, they are required to pass standard physical and psychological assessments used for all military personnel. Upon acceptance, MCs undergo a ten-week training program at the Royal Military Academy where they acquire foundational knowledge of military structures, culture, and operations, as well as the basic competencies needed to function effectively within the military environment.^6^

## Research design and methods

As to date, there is little empirical research that describes the Dutch military chaplaincy practice of one-on-one counseling in relation to moral injury we adopted a grounded, longitudinal qualitative design. In our case, this meant that we collected data of each veteran-MC dyad during 3– 12 months in the form of conversation reports. Moreover, we organized quarterly group meetings with the participating MCs to discuss and process the different stages of the research. This procedural approach allowed us to trace how MCs address moral struggles of veterans in one-on-one meetings and how these meetings evolved over time (Thomson and Holland, 2003).

Data was collected by the first author between October 2022 and April 2024. This section outlines the participants and sampling strategy, collection methods, and the analytical procedure. Ethical considerations are discussed in a dedicated section below.

### Participants

This study involved six MCs who were recruited by the MC coordinator at the Netherlands Veterans Institute (NLVi). At the time of the research, this group represented all Dutch MCs employed by the NLVi who were engaged in one-on-one spiritual counseling with post-active veterans. We describe the participating MCs at the group level to provide context for our findings, while limiting the risk of traceability.

The participating MCs represent the following denominations: Humanist (*n* = 2), Catholic (*n* = 2), and Protestant (*n* = 2). One participant is female; the remaining five are male. All MCs express a critical stance toward the standardization of chaplaincy practice. This is reflected in their educational backgrounds, which include theological and philosophical studies, but no specific additional training in existing chaplaincy interventions. Three participants hold a Master's degree in theology, two in humanistic studies, and one in chaplaincy from a theological university. Before becoming MCs, three gained experience in parish work outside the Dutch armed forces, including hospital chaplaincy and work in the social domain. One MC previously served as a soldier before entering chaplaincy.

Within the Dutch Ministry of Defence, MCs may hold the same position for a maximum of six years. As a result, all participating MCs have held various roles prior to engaging in one-on-one spiritual counseling with post-active veterans, including providing moral education and working at military training bases. Five MCs have been deployed on one or more missions as chaplains, including to Afghanistan, Iraq, Turkey, and Jordan, serving in different branches of the armed forces.

Due to retirement and changes in position, not all MCs participated throughout the full study period. Two MCs joined the project after 10 months, while one MC only participated in the initial 6 months. All six MCs participated in the group meetings, while three MCs (1 Catholic, 1 Humanist, 1 Protestant) also submitted individual conversation reports. The insights of this research are based on the practices of all six participating MCs.

#### Description of the veterans who sought moral injury support from the MCs

We collected data from dyads involving the participating MCs and 12 veterans. The group of veterans was diverse in terms of mission history, covering a broad range of deployments and potentially morally injurious events. Missions ranged from early UN operations in Lebanon and the UNPROFOR in former Yugoslavia to the International Security Assistance Force in Afghanistan.^7^ The veterans all experienced moral injury, as indicated by the Moral Injury Event Scale (Nash et al., 2013; De Goede et al., 2022). Moreover, each had received, or was still receiving, therapy for PTSD-related symptoms at the time of the study. The duration of such trauma-related therapy in civil or military care services ranged from one to eight years. In addition, the sample included both veterans who had only recently established contact with an MC and those who had been in longer-term pastoral relationships, allowing us to capture one-on-one counseling at different stages of development. For more details, see Mudde et al. (2025).

### Data collection

Our qualitative longitudinal research design consisted of two main data sources: conversation reports made by the MCs and quarterly meetings with the whole group of participating MCs.

#### Conversation reports

We offered the MCs an open format without pre-set categories or definitions, that allowed them to write freely about their meetings with the veterans. To help the MCs in offering rich and grounded narrative data about their practice, we discussed the goal and set-up of the research extensively in the first quarterly group meetings (described below). Moreover, we discussed that the reports could be delivered either as written reflections or as audio recordings, and that they should report directly after meeting with a veteran.

The conversation reports included information on the following elements: the location and duration of the meeting, the general atmosphere of the encounter, detailed descriptions and interpretations of the themes addressed in the conversations, the intentions of the MCs, and any unforeseen events. To minimize bias in the self-reporting process, we undertook two measures (see e.g., Dodd-McCue and Tartaglia, 2010; Montonye and Calderone, 2010). First, the first author conducted reflective interviews by phone with the MCs, which served to enrich and clarify each report. This process also ensured that each report contained information on all the elements mentioned above. Second, we collected data on the veterans' perspectives on their conversations with the MCs (Mudde et al., 2025). Each MC submitted between two and seven reports per veteran, resulting in a total of 50 reports.

#### Quarterly group meetings

Every 3 months, we held group sessions with all six MCs. These meetings functioned as collaborative forums in which we collectively discussed and reflected on the different stages of the research process, from participant selection to analysis and interpretation. In total, eight meetings were organized between June 2022 and March 2024, each lasting approximately 120 min. These meetings served several purposes. Initially, they focused on practical aspects of the study, such as participant selection and documenting practices. As the project progressed, the group sessions became important for member checking, thematic validation, and collective reflection, which contributed to the validation of the study (Motulsky, 2021; Urry et al., 2024; Lincoln and Cuba, 1985)

We excluded the meeting reports in the coding process, as we aimed to focus on the direct reflection after each conversation. However, we included the meeting reports in the interpretative steps of the analysis process giving context to the individual reports.

### Analysis

Our analytical approach combined cross-sectional inductive thematic analysis with a longitudinal process perspective. The cross-sectional analysis offered insight into the different ways MCs respond to what veterans with moral injury share. In turn, the longitudinal analysis provided a view of how these conversations evolved over time.

#### Cross-sectional thematic inductive analysis

We used Atlas.ti software to conduct an inductive thematic analysis, which allows themes to emerge from the data without imposing predefined categories (Braun and Clarke, 2021; Naeem et al., 2023). This approach typically begins with several rounds of open coding aimed at familiarization with the data and staying close to the language and meaning of the participants. In our case, this process led to the development of 137 initial codes. These codes varied widely: some described the moral struggles of veterans (e.g., “*reflection on the veteran's repugnance to value his participation in the mission”*), others related to contextual features (e.g., “*meeting at a military barrack”*), and others captured MCs' techniques (e.g., “*mirroring the veteran's behavior,” “deliberate self-disclosure”*).

After this step, we organized the initial codes into eight broader thematic categories (e.g., *conversational techniques, description of the encounter*). This helped us to bring greater clarity to the variety of codes and to understand more precisely how they informed our analysis of how Dutch MCs address experiences of moral injury. To deepen this stage of analysis, we drew on the theory of Dutch military chaplaincy practice as outlined in the introduction. This abductive approach—an iterative process in which theory and empirical data mutually inform and enrich one another (Vila-Henninger et al., 2022)—led us to identify five overarching themes that guided our central research question:

- **Presence** (notes about the nature and dynamics of the relationship, location and duration of the meeting)
- **Embedded Independence** – **in the Dutch Ministry of Defence** (notes about the institutional positioning of the MC within the Dutch Ministry of Defence, e.g. sanctuary, critical stance)
- **Embedded Independence – in interdisciplinary care** (notes about how MCs position themselves toward other care professionals and care paradigms)
- **Pathways of moral reflection – what** (reported conversation topics and goal orientations e.g. helping to tell the story, supporting reconnection with the world)
- **Pathways of moral reflection** – **how** (reported conversational techniques and purposeful actions e.g., facilitating storytelling by using artifacts, attention to the veteran's social network)

#### Code co-occurrence analysis

To further interpret the data, we analyzed whether different types of underlying moral questions informed the MCs' interventions. We used the three underlying moral questions we identified in Mudde et al. (2025) – questions about mission related events, the good life, and the ethical conduct of the Dutch Ministry of Defence - to label relevant segments of the MCs' reports according to the dominant moral theme. This included direct references to one type of the underlying moral questions (e.g., “*I try to make him look at the incident from his civilian identity…”* [Catholic MC and Peter, conversation report 19 September 2023] and indirect references (e.g., “*He has difficulty taking up space in the world, so I developed exercises for him to practice this physically”* [Humanist MC and Joris, conversation report 12 July 2023]. After we used the code co-occurrence tool in Atlas.ti to examine which codes frequently appeared together. This helped us identify interrelated patterns. The interrelation between the moral questions and the identified pathways of moral reflection (what and how) is presented in the results section: pathways of moral reflection. We organized our interpretation of the data using existing chaplaincy taxonomies (Massey et al., 2015; Nash et al., 2018).

#### Longitudinal process analysis

The longitudinal lens allowed us to move beyond the content of conversations to examine how interactions between veterans and MCs evolved over time. To guide this, we developed a tailored analytical framework based on Thomson and Holland (2003).

For each reported conversation, the first and second author created an initial interpretative note addressing how the contact started, where meetings took place, what was discussed and in what manner, how the moral dimensions of the veteran's struggles were addressed, and whether a ‘breakthrough moment' occurred.

Sequential review of the notes enabled us to produce a second-level interpretative note for each veteran–MC dyad, tracing how the relationship developed and how the MC's approach evolved over time. At this stage we addressed questions of development, continuity, and change: How did the veteran's initial need evolve across meetings? Did the location and duration of meetings shift? Which dimension of military chaplaincy practice (presence, moral reflection, embedded independence) tended to dominate? We also examined how the topics introduced in the conversations related to one another, whether certain elements recurred, how the relationship itself developed, and at which points breakthrough moments emerged. Finally, we noted whether and how trajectories came to a close, and how the manner of closure reflected the preceding development.

By systematically comparing these second-level notes across all cases, we were able to move beyond individual trajectories and identify shared developmental patterns. This comparative analysis formed the basis for four typologies that capture recurring forms of contact between veterans and MCs.

### Ethical considerations

Dutch MCs have pleaded absolute confidentiality. This research, which required MCs to reflect on their work with specific veterans, posed a potential conflict with this professional norm. This issue was extensively discussed during the project's initiation with participating MCs and the office holders. The motivation to proceed with the study was based on the limited existing research on the day-to-day practice of MCs—particularly in secular contexts—and the growing relevance of their role in responding to moral injury.

Together with the MCs, we decided to resolve the potential conflict with the MCs' plea to confidentially by prioritizing the veterans' informed consent. Therefore, the ethical framework for this study was grounded in the principle that the veterans' consent would be the guiding criterion. To do so, veterans received an information letter and verbal explanation at the time of their first interview with the first author. They were explicitly asked to give permission for the MCs to share the general outline of their meetings and were reminded that they could withdraw their consent at any point without the need for justification. The MCs verbally consented to participating in this research project during the first group meeting in which we introduced the research project, and we checked for consent at every quarterly meeting and again before the publication of this article. Prior to this group meeting we already had approvement for this research project from the NLVi.

To uphold the veterans' consent over time, MCs regularly checked in with veterans about their continued participation. Additionally, the MCs cross-checked with the first author what veterans had shared in their interviews, ensuring they did not disclose more than what veterans themselves had already revealed. During the research project it appeared that the participating veterans were in fact very willing to participate. Even more so, some of them even shared their story with their real name in the context of an exhibition^8^ we organized to share the topic of moral injury with a wider audience. For these veterans, sharing their story and struggles within the context of the research and exhibition was also a form of recognition.

## Results

In the results section, we first present the findings from the cross-sectional analysis, identifying three core dimensions of military chaplaincy practice in the context of moral injury: presence, embedded independence, and moral reflection. We then explore the dimension of moral reflection in more depth, outlining the different pathways of moral reflection MCs follow to address the underlying moral questions of veterans and how these conversations unfold over time.

### Cross-sectional analysis: three key dimensions of military chaplaincy practice

**Figure 1:**
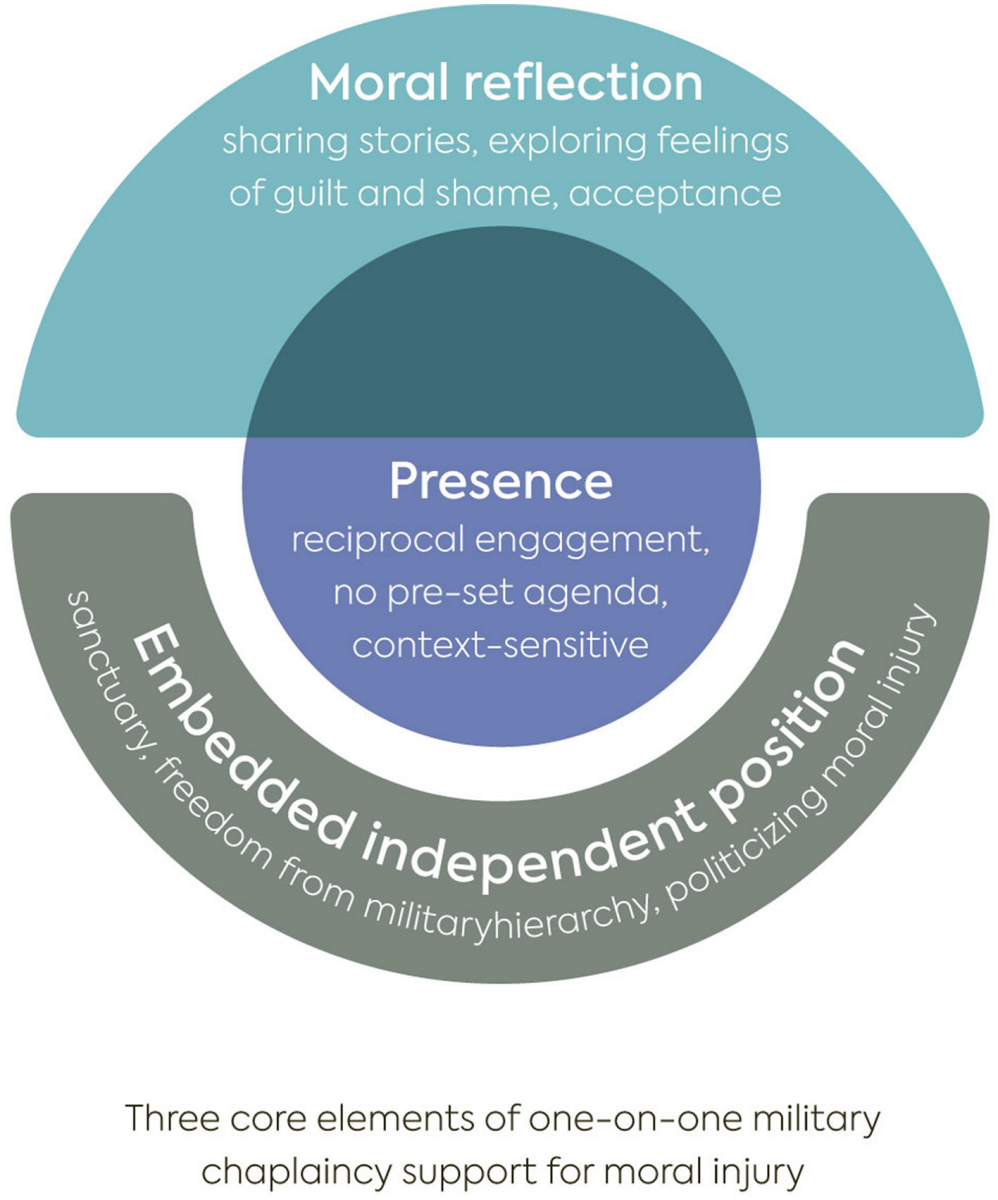
Three core elements of one-on-one military chaplaincy support for moral injury.

The cross-sectional analysis brought to the front that MCs work in three interrelated dimensions in their one-on-one counseling with veterans experiencing moral injury. The heart of the conversation is the relational dimension, grounded in the encounter. We characterize this dimension as ‘presence,' capturing both the physical context (such as location and absence of time pressure) and the relational quality between the MC and the veteran. Second, the institutional dimension reflects the unique position from which MCs operate, which we term as an embedded independent position. MCs practice this position within the Dutch Ministry of Defence and the National Healthcare System for Veterans. Third, moral reflection unfolds through the encounter, nested within the MCs' embedded independent position. This dimension refers to the reflective support MCs offer in helping veterans process moral struggles and includes their goal orientation, the topics discussed, and the conversational techniques they employ. This led us to propose the following model (see Figure 1) of military chaplaincy practice in addressing moral injury in the Dutch context.

In the following section, we elaborate on the three interrelated dimensions of this model and how various moral questions are addressed by the MCs over time.

#### Presence

Conversation reports and quarterly meetings with the MCs reveal that much of their work centers on presence—a way of working without a pre-set agenda, marked by attentiveness to contextual meaning and grounded in reciprocal engagement. MCs enact this approach by devoting considerable time and attention to the temporal, physical, and relational dimensions of their encounters with veterans.

Considering the temporal dimension: MCs report that they do not follow a preset agenda. Instead, conversations unfold according to what is most pressing for the veteran in that specific moment, time, and place. There are no fixed time limits or predetermined number of sessions. MCs and veterans are free to shape the frequency and duration of their contact as the process evolves. In some cases, this flexibility is made explicit to the veteran:

“*He also asked at one point, “So, how many sessions do we have left together?” I explained to him that this isn't how we work. We don't work with fixed sessions—there doesn't necessarily have to be an end to it. For the time being, we can keep meeting. That sense of timelessness is something he found very comforting; it makes you, in the end, a truly reliable conversation partner.”* [Humanist MC and Frank, report 4 October 2023]

Physically, the meeting location is often determined by what is meaningful for the veteran. Encounters may take place at home, during a walk, or at a military barrack.

Notably, the relational dimension is not treated merely as instrumental—a way to build rapport before ‘the real work' begins. Instead, the encounter itself is seen as meaningful and valuable and an end in itself. MCs emphasize reciprocal engagement, often sharing their own thoughts and experiences to foster a more equal and authentic relationship:

“*And besides that, he also really enjoys—this took up a large part of our conversation—that I share things about myself: what I do, what I'm working on, a bit from my private life. He clearly has a need for what you might call ‘normal' contact, something almost like a friendship, where I also talk about my own daily struggles, vacations, that kind of thing. I notice it really lifts his spirits—just receiving that kind of everyday, ordinary input.”* [Humanist MC and Frank, report 26 May 2023]

These three elements – time, location, and relation – serve not only as preconditions for building trust, allowing the veteran to feel safe enough to share their story. Ultimately, it is the encounter itself that can be morally formative. For instance, the relationship with the MC may help a veteran re-establish self-trust and regain faith in the goodness of others. Furthermore, the choice of meeting location can also carry symbolic or moral significance—for example, meeting at a military barrack may offer a sense of recognition and affirmation of one's identity as a veteran.

#### The embedded independent position of the MC

MCs position themselves within their broader institutional contexts from what can be described as an embedded independent stance—a unique ‘in-between' role. The data reveal that MCs enact this embedded independence in relation to both the Dutch Ministry of Defence and the National Healthcare System for Veterans.

##### Navigating embedded independence in the Dutch Ministry of Defence

The reports show how MCs use their embedded independent position to engage with veterans in ways that affirm moral complexity and acknowledge institutional shortcomings. A first way this critical stance becomes visible is in how MCs conduct their own inquiries into incidents raised by veterans. They independently explore the operational and institutional contexts of specific missions to better understand the dynamics underlying morally troubling events. Based on these investigations, MCs form their own perspectives and bring these into the conversation—not to impose an interpretation, but to foster a constructive and critical dialogue with the veteran.

Second, MCs do not hesitate to explicitly acknowledge institutional failures, once they have gained a thorough understanding of the situation. This can serve to validate the veteran's moral experiences and affirm their sense of justice, particularly when institutional recognition has been absent elsewhere.

“*I gave Walter full recognition that something went wrong in this situation. I'm aware that, in my role, I represent both the institution of the military and the institution of the Catholic Church. In acknowledging that mistakes were made, I choose not to stand with the institution—but with the victim*.” [Catholic MC and Walter, report 21 December 2022]

Third, MCs also act as representatives of the Dutch armed forces, but they do so from a critical and morally attuned position. This representational role can take the form of practical care—such as intentionally seeking out individuals during return visits to former conflict zones—or symbolic gestures, such as naming moral wrongdoing or offering institutional acknowledgment on behalf of the military.

Finally, a defining feature of the MC's institutional role is their position of confidentiality. Veterans are explicitly informed that conversations with MCs are not shared within the organization. This guarantees a protected space in which veterans can speak freely about their experiences—including feelings of betrayal, doubt, or mistrust—without fear of institutional consequences.

As with the relational dimension of presence, this embedded independence fosters trust and openness in the conversation. At the same time, it can also have moral significance in itself—for instance, by validating a veteran's experience of betrayal by the military institution.

##### Critical positioning in interdisciplinary care

The MCs' critical positioning within the National Healthcare System for Veterans manifests in two primary domains: in their conversations with veterans and in their interactions with other care professionals.

All veterans in this study also received trauma therapy for PTSD (-related) symptoms. As a result, MCs regularly reflected with veterans on these therapeutic trajectories, particularly where the addressed events or symptoms were closely intertwined with experiences of moral injury. These reflections vary widely. In some cases, MCs actively support the therapeutic process—for example, by helping veterans prepare for meetings with a psychiatrist, such as collecting photographs together, or by revisiting themes that emerged during an EMDR session to explore them in more depth afterward.

At the same time, MCs attend to veterans' concerns, doubts, or reflections about therapy. Veterans may, for instance, express anxiety about the emotions therapy might surface, or reflect on how therapy affects their sense of identity. Conversations with MCs often become safe spaces where veterans can explore such feelings, question aspects of their treatment, or share ambivalence. Two characteristics underpin these discussions: first, the veteran's input always leads the conversation; MCs do not initiate critique but respond to what the veteran brings. Second, these conversations occur within the MCs' sanctuary, reinforcing the safety of the space for open reflection.

In their professional role within the broader care system, MCs voice a more explicit and critical stance. A recurring theme is the concern about a potential over-medicalization of moral injury. Importantly, MCs do not aim to undermine the work of trauma professionals; rather, they offer complementary moral and relational perspectives on veterans' experiences. Reports show that MCs express these concerns in a constructive and dialogical manner—for instance, in interdisciplinary meetings or informal consultations—where they challenge dominant diagnostic paradigms and offer alternative moral or relational perspectives on veterans' symptoms:

“*The social worker was the first to tell him that he has PTSD. The usual line of thinking is that the dismissal has triggered memories of his war experiences, but I believe it's actually the dismissal itself that is troubling him. He served with the medical troops —was always ready, day and night—but when it really mattered, he was cast aside, and the Dutch Ministry of Defence failed to keep its promises. I also voiced this perspective in meetings and conversations with involved care professionals. There is a certain temptation to foreground the war—because that sounds more serious.”* [Catholic MC and Simon, reflective phone call 10 May 2023]

In conclusion, MCs' embedded independence within the care system enables them to continuously foreground the moral and existential dimensions of veterans' experiences—both in their conversations with veterans and in their interactions with care professionals.

#### Pathways of moral reflection

The code co-occurrence analysis provided insight into the various approaches MCs adopt in response to different types of moral questions.

Earlier we identified three underlying moral questions that underpin experiences of moral injury: questions about mission related events, questions about the good life and questions about the ethical conduct of the Dutch Ministry of Defence (Mudde et al., 2025). The analysis through code co-occurrence shows that the MCs attune their responses to each of these questions—not only in terms of the perceived need and intended purpose of their support, but also in the approach they choose. During the analysis process, a distinction emerged between pathways MCs choose when moral struggles are directed inward (questions concerning goodness in the self) and to those directed outward (questions about goodness in the world), resulting in different pathways for four types of moral questions. However, we found no clear pattern in how MCs respond to different types of morally injurious events—such as acts of commission, omission, or witnessing inhumane acts. One possible explanation is that the MIES scores of the veterans indicate that most are grappling with a mixture of morally injurious experiences, making it difficult to isolate a single dominant type.

Table 1 below summarizes the outcomes of the code co-occurrence analysis. It shows the different pathways of moral reflection [combined goal orientations (to what end?) and the corresponding approaches (how?)] that MCs adopt in response to four distinct types of moral questions. It is important to note that, in practice, moral questions are often intertwined, resulting in overlap and dynamic interplay between approaches. Still, the aim here is to demonstrate that MCs adopt distinct focal points in response to the different types of moral struggles that veterans bring into the counseling relationship. After introducing the Table 1, we elaborate on the content of each column and the relation between the goal orientation and corresponding approach of the MCs.

**Table 1:** Pathways of moral reflection in military chaplains' one-on-one support for veterans with moral injury.

**Addressing moral questions about mission related events**	**Addressing moral questions about goodness in self**	**Addressing moral questions about goodness in the world**	**Addressing moral questions about the Dutch Ministry of Defence**
**To what end?**	**To what end?**	**To what end?**	**To what end?**
Helping to tell the story so that the veteran is not left alone with it	Assisting in recognizing veterans' own victimhood	Stimulating to reconnect with the world	Assisting in recognizing veterans' own victimhood
Review certain events to make them more bearable	Releasing the perceived need for continued atonement	Helping to set boundaries to veterans' moral responsibility	Helping to critically engage with the Dutch Ministry of Defence
Giving recognition for victims of war	Creating a more complex and diverse view on feelings of guilt and shame		
**How?**	**How?**	**How?**	**How?**
Facilitating storytelling by using artifacts (photographs, military artifacts, maps)	Exploring themes of guilt and shame	Attending to the body and cultivating greater physical awareness and relaxation	Acknowledging that the military has made mistakes
Introducing alternative perspectives to help the veteran zoom out, reframe the situation, or place an event within their broader life narrative	Examining the veteran's multiple identities as civilian and service member	Taking walks together and explicitly paying attention to the (beauty of the) world	Reflecting on the difficulty of feeling recognized and appreciated for participation in a certain mission
Underlining the social dimension of moral injury: you don't bear this alone	Acknowledging the progress a veteran is making	Engaging in dialogue about everyday events	Underlining the social dimension of moral injury: you don't bear this alone
Reflecting on the social reception of a certain mission	Normalizing setbacks	Reflecting on current sociopolitical developments	Exploring and opening up discussions around the underlying values and meaning of certain experiences and events
Discussing the suffering of civilian victims of war	Emphasizing that the veteran retains agency in shaping their own life	Exploring moral themes and questions of goodness in the world through specific (Biblical) narratives offered by the MC, in alignment with the veteran's own worldview or spiritual beliefs	
Looking out for certain people in the (post) conflict area	Deliberate self-disclosure of the MC	Reflecting on challenges in setting boundaries around responsibility	
	Attention to the veteran's social and relational network		

Column one: when veterans face moral questions related to actions during their missions, MCs support them in sharing their stories so they no longer carry the burden alone. In addition, MCs offer and acknowledge recognition for the victims of war. This process involves joint reflection on specific events, incorporating multiple perspectives for a more nuanced and multifaceted understanding of responsibility and guilt. Throughout these conversations, MCs emphasize and explain the societal dimension of moral injury.

“*I think of the girl who died in Jan's arms. He saw her—and because of that, she did not remain a nameless victim. But Jan does not carry this alone; we all carry it… That's why it is so important not to reinterpret or reframe this too quickly in therapy—because in doing so, we deny these victims the recognition of their suffering…. This guilt must be carried, because it forms part of our collective conscience. I believe we are often too quick to forgive, too quick to absolve guilt. The aim should not be, first and foremost, to make the veteran feel better—because that renders the victim secondary. It also individualizes the burden, when in fact it should be shared*.” [Catholic MC and Jan, reflective phone call 5 April 2024]

Column two: in cases where veterans struggle with questions about goodness in the self, MCs aim to help them recognize their own victimhood and release the belief that ongoing atonement is necessary. They do so by encouraging greater awareness and appreciation of the veteran's civilian identity, by mirroring progress and normalizing setbacks, and by emphasizing that the veteran retains agency in shaping their life.

“*Freek has something compulsive about him—he's stuck in causal thinking. That can make conversation difficult at times. During our talks, he often says: “I'm very religious, but there's also a Satan who can whisper things to you.” The social worker sees his belief in destiny and such as a compulsive tendency that needs to be treated, but I try to connect with him through the religious dimension. I emphasized that human beings have free will. God could have created humans without it—but He deliberately placed the tree of the knowledge of good and evil before us.”* [Catholic MC and Freek, conversation report 9 June 2023]

Column three: when veterans struggle with questions about the goodness of the world, MCs seek to strengthen the veteran's connection to the world, while also helping them set clearer boundaries around their sense of moral responsibility. MCs work toward these goals by engaging as equal conversational partners, reflecting on everyday experiences, the veteran's moral burden, and broader political or societal issues. In doing so, they also share their own perspectives, helping to interrupt negative moral reasoning and open space for new insights.

“*Yesterday we didn't go for a walk; instead, we sat on his terrace overlooking the water. There were grebes with chicks on their backs—that's the kind of thing you talk about. Of course, there's a deeper intention behind that: in this case, the beauty of nature as a way to explore whether he is open to experiencing a sense of meaning—through the beauty of the world. It also turned into a fairly philosophical conversation. We spoke at length about religion, and the influence of religion on public life—Christianity, Islam. He's quite preoccupied with these things... We actually had a proper debate. I didn't always agree with him, and that led to a really pleasant and engaging exchange of views. I personally found it a rewarding conversation as well. He also said it did him good to engage on that more philosophical level.”* [Humanist MC and Frank, conversation report 04-10-2023]

Column four: when veterans doubt the ethical conduct of the military institution, MCs again help them recognize their own victimhood—a goal orientation that overlaps with responses of MCs to questions about goodness of the self (column 2). Additionally, MCs engage veterans in critical reflection on the military organization itself. These conversations explore the underlying values and meanings of the veteran's experiences, and often include acknowledgment of institutional failure. MCs also address the veteran's struggles with feeling recognized or valued for their service, and, once again, emphasize the collective and societal dimension of moral injury.

“*Simon's Military Retirement request was denied. As far as I know, he's still in the process of piecing his story together. In the meantime, I've been thinking about possible avenues for him to share that story and receive some form of recognition—but I haven't yet found an answer. In our conversation, I focused more on the emotional level: acknowledging what happened, acknowledging that he is ill or vulnerable. I asked questions like: Is he angry with the military? Does he feel the military owes him something? Questions about guilt and responsibility. But also: what is it like for him to offer help to others? And why is it so difficult for him to accept help himself?”* [Catholic MC and Simon, reflective phone call 10 January 2024]

#### Four typologies of contact development

The longitudinal analysis provided more insight into how the meetings between veterans and MCs developed and how different pathways of moral reflection can unfold. It first of all reveals that MCs consistently engage with all three dimensions of their practice—presence, embedded independence, and moral reflection—throughout the entire trajectory. This indicates that the dimensions are not sequential but interwoven from the outset.

Second, it became clear that many factors shape how conversations between MCs and veterans evolve over time, including the specific moral or existential struggles of the veteran, the counseling style of the MC, the degree of spiritual alignment between them, and how the encounters related to concurrent trauma therapy. Because of this diversity, we did not find robust patterns in the unfolding of specific aspects such as relational trust.

Nevertheless, when comparing trajectories at a higher level, consistent developmental patterns emerged. We synthesized these into four typologies that represent distinct ways in which the meetings between veterans and MCs developed over time:

1. **Clear request and alignment:** The veteran expresses a clear need or request, and the MC's guidance corresponds effectively. Sessions often focus on reflection around a specific event, and once a form of closure is reached, the trajectory comes to a natural conclusion.
2. **Emerging focus:** The initial request or need is less specific, but over time, central themes emerge and are developed consistently throughout the sessions. Once these have been sufficiently addressed, the contact also reaches a natural conclusion.
3. **Development through the relation:** The veteran's needs remain ill-defined, and themes tend to repeat. The trajectory evolves primarily through the growing relational bond between veteran and MC. These trajectories are typically long-term.
4. **Minimal responsiveness, story-sharing focus:** As in type 3, the request or need is vague and themes repeat, but in this case, the veteran shows limited responsiveness to the MC's interventions. The MC focuses primarily on maintaining the relationship. These trajectories are also long-term.

In seven cases, the longitudinal analysis revealed that MCs reported a **breakthrough moment**. These refer to pivotal moments in the development of their conversations, for example when a veteran for the first time shared a crucial element of a story, or when an important shift occurred either in the lives of the veteran or in their encounters:

“*At least now we've found a thread to follow. We've touched on this before, of course, and I know this is a very sensitive issue for him. But we've now reached a point where we can really pursue it. In the past, he always tended to avoid it a little, but now I noticed he became very emotional—and those emotions were open for discussion. There was space to reflect on them.”* [Humanist MC and Joris, report 12-09-2023]

Such breakthroughs only occurred after sustained engagement and were absent in type 4 trajectories. Finally, the longitudinal analysis suggested that veterans grappling with fundamental questions of goodness—whether concerning themselves or the world—were most often situated in types 3 and 4. These profound moral and existential struggles appear harder to articulate and require extended relational work, patience, and trust-building to be adequately addressed.

## Discussion

This study aims to provide a qualitative, longitudinal perspective on how Dutch MCs engage in one-on-one meetings with veterans experiencing moral injury. In the discussion, we elaborate on how our findings contribute to existing literature on the role of MCs in addressing moral injury, structured around the three core elements presented in Figure 1.

### Creating moral space through presence

Our study shows that presence—an intentional, relational practice of being fully with veterans—plays a formative role in how the moral dimensions of morally injurious experiences are addressed within one-on-one spiritual counseling by Dutch MCs. The centrality of the ministry of presence in chaplaincy practice is widely acknowledged in the general chaplaincy profession (Adams, 2019; Lawton and Cadge, 2024), and in military chaplaincy (Grimell et al., 2025; Liuski and Ubani, 2021; Pleizier and Schuhmann, 2022). This study offers an additional perspective by emphasizing the moral significance of presence, particularly in addressing veterans' experiences of moral injury.

Our findings emphasize the moral value of the MC's ability to meet without a pre-set agenda or fixed time constraints. The opportunity Dutch MCs have to meet with veterans without such temporal limitations often contrasts sharply with other forms of trauma therapy veterans receive or have received. Moreover, this study highlights the moral value of Dutch MCs' ability to meet veterans at locations that hold personal significance or where they can engage with the beauty of nature. The choice of meeting place can carry symbolic or moral significance—for example, meeting at a military barrack may offer a sense of recognition and affirmation of one's identity as a veteran. In inter- or non-religious contexts, opportunities to engage in horizontal transcendence—e.g. experiencing the beauty of nature—can be particularly valuable in helping veterans to learn to see goodness in the world again (Berghuijs et al., 2013).

Finally, our study sheds light on the moral significance of the relational dimension of military chaplaincy practice in addressing moral injury. As detailed in the results, the relationship with the MC may help a veteran to re-establish self-trust and regain faith in the goodness of others. This is particular relevant with struggles that are difficult to voice, as suggested in our longitudinal analysis. Theories of attentiveness in care emphasize that the relational work MCs engage in is not merely instrumental but can be an end in itself (Baart, 2001; Klaver and Baart, 2011). Our study demonstrates how this relational attentiveness functions within the context of moral injury. The intersubjective space created by MCs can be understood as a ‘moral space' where veterans can once again experience ‘goodness' in human-to-human relationships. This experience may meaningfully contribute to a restored sense of goodness in both self and world.

### Military chaplaincy work in relation to moral injury is politicized

Our findings show that one-on-one counseling for moral injury is deeply rooted in the unique institutional position of Dutch MCs and resonates with other European military chaplaincy models in which MCs are embedded in the organization yet are self-directed (Liuski and Ubani, 2021; Liuski, 2023). We characterize this position as one of embedded independence, a term that captures the paradoxical position and underlines how the MCs' embeddedness significantly informs and colors their independence. The study's results show that this position enables Dutch MCs to politicize the experience of moral injury in two ways.

The first way comes into practice in the internal function of MCs' embedded independent position. MCs offer a sanctuary and create ‘moral space' in which veterans can speak openly and receive responses that are informed by military culture, but not governed by organizational interests. In doing so, MCs emphasize that the primary source of moral injury is not individual but sociopolitical (see also Hamidi, 2023), helping veterans recognize the social responsibility in the emergence of morally injurious events and thereby politicizing their experiences.

Second, the position of embedded independence also has an externally directed function, where it may foster greater institutional sensitivity to moral concerns and questions of human dignity. Here, MCs create ‘moral space' within the institutional domain to voice issues of moral concern. This is the political role MCs take up in addressing moral injury. As our results show, they do this in the institutions of the Dutch Ministry of Defence – where they highlight the suffering of civil victims of violence in the stories of the veterans out of a commitment to justice. But also in the context of the National Veteran Health Care System, where they make a case for not (overly) medicalizing experiences of moral injury. Other authors have similarly brought attention to the contribution of MCs in challenging dominant medical models in veteran care (Grimell, 2024b) and foregrounding the spiritual and moral dimensions of moral injury in interdisciplinary care (Antal et al., 2023; Carey et al., 2024; Smith-MacDonald et al., 2018; Meador and Nieuwsma, 2018). Our study provides empirical insights into how Dutch MCs practice this in one-on-one counseling with veterans who experience moral injury.

### Layered and variably evolving moral reflection

Many of the approaches we identified in Table 1 resonate with the phases of existing military chaplaincy interventions, as outlined in the introduction. These include, for example: establishing rapport, reflecting on and reviewing morally injurious events, addressing feelings of guilt, and working toward reconnection with significant others or spiritual sources. This study adds the following complementary and challenging insights:

First, our findings on the moral reflection in which MCs and veterans engage show how different approaches of MCs align with specific underlying moral questions. This finding resonates with the observation by Drescher et al. (2018) that questions about mission-related actions comprise only a small part of the overall military chaplaincy practice in addressing moral injury. Our aim in structuring these findings in the table we present, is to bring nuance and complexity to our understanding of how MCs attune to veterans' experiences of moral injury; their feelings of guilt, shame and betrayal. It illustrates how MCs attune their approach to these specific questions—whether they concern personal responsibility, trust in others, or broader notions of justice. By highlighting these variations, the table provides insight into the complexity of moral injury and illustrates the different ways MCs can respond in a meaningful and context-sensitive manner.

Second, our study raises questions about directionality in the development of chaplaincy conversations: are they primarily shaped by a ‘pull' toward a goal orientation, or ‘pushed' by the underlying struggles? While this may appear to be a chicken-and-egg dilemma, these, sometimes subtle, shifts in intention could be particularly significant in the context of moral injury. Therefore, rather than structuring the table according to intended effects, methods, and interventions as seen in existing chaplaincy taxonomies (Massey et al., 2015), we deliberately organized it around the interplay between underlying moral struggles, goal orientation, and concrete actions to underline how underlying questions significantly shape the approach of the MCs.

Third, the longitudinal perspective of our study highlights that there are multiple ways in which the contact between MC and veteran can unfold. Lastly, our study underlines that it may a considerable amount of time to be able to adequately address experiences of moral injury, especially when veterans are struggling with questions about goodness in the world or the ethical conduct of the Dutch Ministry of Defence.

### Limitations and directions for future research

This study aims to shed light on the practice of MCs working in the Netherlands, particularly in one-on-one meetings with veterans experiencing moral injury. It shows how their practices are rooted in presence and embedded in a unique institutional position, which inherently politicizes the work they do around moral injury. Nevertheless, this study is limited by its small sample size. Future research should explore whether the model, pathways of moral reflection and typologies proposed here resonate with the broader population of MCs in the Netherlands.

Our findings also highlight the importance of further exploring the longitudinal dimension of the military chaplaincy practice. While we were able to identify broad patterns in how conversations developed over time, more focused data would be needed to understand how key relational aspects—such as trust—emerge and evolve. Given the centrality of the relationship, this is a promising area for future research. Another valuable avenue would be to investigate which specific professional actions precede breakthrough moments in the counseling process.

Lastly, while considerable international research has examined collaborative programs between MCs and mental health professionals (see e.g., Nieuwsma et al., 2024), the Dutch context is different. Due to their sanctuary, MCs in the Netherlands do not report to other care providers. Yet, our study shows that they are aware of other treatments veterans receive, they have contact with other care professionals in interdisciplinary meetings, and try to align their conversations accordingly. This raises important questions about whether and how Dutch MCs and mental health professionals might further coordinate their approaches—a topic worth exploring in future research.

## Data Availability

The datasets presented in this article are not readily available because the small participant sample and longitudinal research design make it difficult to guarantee full anonymization. However, the metadata supporting the conclusions of this study will be made available by the authors upon reasonable request. Requests to access the datasets should be directed to Laura Mudde, laura.mudde@uvh.nl.

## Footnotes

^1^Viering 60 jaar Humanistisch Geestelijke Verzorging bij Defensie. Available online at: https://www.dgv.nl/actueel/nieuws/2024/09/01/index [Accessed June 14, 2025].

^2^This article builds on and complements a companion study published in this special issue, which analyzed the perspective of post-active veterans on their moral struggles and their experiences of meeting with an MC (Mudde et al., 2025). While that article focused on veterans' lived experiences, the present study shifts the lens to the perspective of MCs themselves.

^3^Visie Geestelijke Verzorging. Available online at: https://www.dgv.nl/over-dgv/visie/visie1 [Accessed June 11, 2025].

^4^Orthodoxe Geestelijke Verzorging bij Defensie. Available online at: https://www.dgv.nl/actueel/nieuws/2024/12/14/index [Accessed May 27, 2025].

^5^Visie Geestelijke Verzorging. Available online at: https://www.dgv.nl/over-dgv/visie/visie1 [Accessed June 11, 2025].

^6^Ministerieel_Besluit_Functionele_Kaders_GV_(getekend).pdf [Accessed June 19, 2025].

^7^Complete list of missions: UNIFIL, United Nations Interim Force in Lebanon (1978–present). UNTAC, United Nations Transitional Authority in Cambodia (1992–1993). UNPROFOR, United Nations Protection Force in former Yugoslavia (1992–1995). ECMM, European Community Monitoring Mission in East/Middle Europe (1991–2007). KFOR, Kosovo Force (1999–2000), and ISAF, International Security Assistance Force in Afghanistan (2002–2011).

^8^Post Arnhem Bearing Witness. Available online at: https://platformpost.nl/en/program/arnhem-bearing-witness-elma-cavcic-nadine-hattom [Accessed June 12, 2025].
